# Nonalcoholic Fatty Liver Disease Is Exacerbated in High-Fat Diet-Fed Gnotobiotic Mice by Colonization with the Gut Microbiota from Patients with Nonalcoholic Steatohepatitis

**DOI:** 10.3390/nu9111220

**Published:** 2017-11-06

**Authors:** Chien-Chao Chiu, Yung-Hao Ching, Yen-Peng Li, Ju-Yun Liu, Yen-Te Huang, Yi-Wen Huang, Sien-Sing Yang, Wen-Ching Huang, Hsiao-Li Chuang

**Affiliations:** 1 Animal Technology Laboratories, Agricultural Technology Research Institute, Miaoli 350, Taiwan; 1042032@mail.atri.org.tw; 2 Department of Molecular Biology and Human Genetics, Tzu Chi University, Hualien 970, Taiwan; yching@mail.tcu.edu.tw; 3 Graduate Institute of Veterinary Pathobiology, National Chung Hsing University, Taichung 402, Taiwan; d106047002@mail.nchu.edu.tw; 4 National Laboratory Animal Center, National Applied Research Laboratories, Taipei 115, Taiwan; wide0720@nlac.narl.org.tw (J.-Y.L.); evan@nlac.narl.org.tw (Y.-T.H.); 5 Liver Center, Cathay General Hospital Medical Center, Taipei 106, Taiwan; yiwenhuang@ntu.edu.tw (Y.-W.H.); jaab@cgh.org.tw (S.-S.Y.); 6 School of Medicine, Taipei Medical University College of Medicine, Taipei 110, Taiwan; 7 Department of Exercise and Health Science, National Taipei University of Nursing and Health Sciences, Taipei 112, Taiwan

**Keywords:** gut microbiota, humanized gnotobiotic mice, nonalcoholic fatty liver disease, high-fat diet

## Abstract

Nonalcoholic fatty liver disease (NAFLD) is a serious liver disorder associated with the accumulation of fat and inflammation. The objective of this study was to determine the gut microbiota composition that might influence the progression of NAFLD. Germ-free mice were inoculated with feces from patients with nonalcoholic steatohepatitis (NASH) or from healthy persons (HL) and then fed a standard diet (STD) or high-fat diet (HFD). We found that the epididymal fat weight, hepatic steatosis, multifocal necrosis, and inflammatory cell infiltration significantly increased in the NASH-HFD group. These findings were consistent with markedly elevated serum levels of alanine transaminase, aspartate transaminase, endotoxin, interleukin 6 (IL-6), monocyte chemotactic protein 1 (Mcp1), and hepatic triglycerides. In addition, the mRNA expression levels of Toll-like receptor 2 *(Tlr2*), Toll-like receptor 4 *(Tlr4)*, tumor necrosis factor alpha (*Tnf-α*), *Mcp1*, and peroxisome proliferator-activated receptor gamma (*Ppar-γ*) significantly increased. Only abundant lipid accumulation and a few inflammatory reactions were observed in group HL-HFD. Relative abundance of *Bacteroidetes* and *Firmicutes* shifted in the HFD-fed mice. Furthermore, the relative abundance of *Streptococcaceae* was the highest in group NASH-HFD. Nevertheless, obesity-related *Lactobacillaceae* were significantly upregulated in HL-HFD mice. Our results revealed that the gut microbiota from NASH Patients aggravated hepatic steatosis and inflammation. These findings might partially explain the NAFLD progress distinctly was related to different compositions of gut microbiota.

## 1. Introduction

Diet-induced nonalcoholic fatty liver disease (NAFLD) is one of the most common chronic liver disorders worldwide [1] due to the epidemic increase in the prevalence of obesity in affluent societies. The development and progression of NAFLD involve complex pathophysiological processes [2] and are influenced by host factors such as genetic polymorphisms, diet, and, as reported more recently, the composition of the gut microbiota. NAFLD includes a wide spectrum of hepatic pathological characteristics ranging from simple steatosis to nonalcoholic steatohepatitis (NASH) with or without fibrosis, cirrhosis, and hepatocellular carcinoma [3]. In a subset of the population (~10–20%), NAFLD progresses to NASH. This population has hepatic steatosis accompanied by inflammation and fibrosis. Although fibrosis is not a requirement for the diagnosis of NASH, it is often present in NASH patients [4]. Moreover, in the liver samples of patients with NAFLD, inflammasome components are significantly upregulated in the patients with NASH when compared to those with non-NASH NAFLD [5].

Recent studies have highlighted the role of the gut microbiota in the regulation of energy homeostasis and revealed its association with a number of disease states such as allergy, inflammatory bowel disease, cancer, and diabetes [6,7,8,9]. Gabele and coworkers have shown that combined administration of a high-fat diet (HFD) and dextran sulfate sodium (DSS) leads to disturbances in the homeostasis of the relation between the gut microbiota and the host; this change may promote bacterial translocation from the gut into the portal circulation, further increasing liver damage [10]. Additionally, Henao-Mejia et al. have provided evidence that modulation of the intestinal microbiota through multiple inflammasome components is a critical determinant of NAFLD and NASH progression [2]. These studies indicate that the gut microbiota is deeply involved in NAFLD and obesity.

Several recent studies have used germ-free mice that were colonized by a human gut microflora to investigate the disease progression of inflammatory bowel disease (IBD) colitis [11] and intestinal physiology [12]. Turnbaugh and collaborators transplanted fresh human fecal microbes into germ-free C57BL/6J mice to create an animal model with a defined and representative human gut ecosystem [13]. The gut microbiota of these humanized gnotobiotic mice yielded responses similar to those of their human counterparts including microbial colonization dynamics, biogeographical distribution, and responses to dietary perturbations [13,14]. Follow-up experiments involving human fecal samples from twins discordant for obesity (when transplanted into gnotobiotic mice) showed significant body composition differences [15]. Based on these findings, we believe that the humanized mouse platform for studies on NAFLD pathogenesis is a powerful tool. Therefore, our aim was to determine the role of the gut microbiota in NAFLD by colonizing germ-free (GF) mice with fresh feces from NASH patients or from healthy individuals. We uncovered a significant correlation between gut microbiota composition and progression of NAFLD and elucidated how these differences may interfere with host metabolism and dietary energy absorption and storage.

## 2. Materials and Methods

### 2.1. Animals

All the animals were housed in sterilized isolators at the temperature of 22 ± 1 °C, 55–65% relative humidity, in a 12-h light/12-h dark cycle. All the procedures were performed at an animal facility accredited by the Association for Assessment and Accreditation for Laboratory Animal Care, International, with the approval of the Institutional Animal Care and Use Committee at the National Laboratory Animal Center (approval number IACUC2011M16R01; approval date 10 September 2012). The methods in this study were carried out in accordance with the approved guidelines.

### 2.2. Microbiota Transplantation and Experimental Design

A total of 40 germ free (GF) mice (10 mice per group) were used in this study. Three- to four-week-old male GF C57BL/6JNarl mice were orally inoculated with a fresh fecal mixture obtained from donors: either healthy humans (HL) (*n* = 10) or NASH patients (NASH) (*n* = 10). Disease status (HL or NASH) was first determined based on serum chemistry (alanine transaminase (ALT), aspartate transaminase (AST), triglycerides (TGs), cholesterol, and fasting glucose) and imaging by abdominal ultrasonography, and then a liver biopsy was performed only on NASH patients for confirmation. The donors of healthy human’s feces had normal body mass index (BMI) and clinical biochemistry, including ALT, TGs, cholesterol (T-CHO), and glucose (GLU) (Table S1).

The average anthropometric variables for the HL and the NASH donors are summarized in Table S1. For each group of participants (the HL and NASH), 1 g of a fresh fecal sample from each of 10 individual human donors was mixed with 9 mL of PBS (1:9), and then the samples were pooled at equal volumes. Fecal mixtures were vortexed for 3 min at room temperature, and 0.5 mL was orally inoculated into each GF recipient mouse by gavage. After 4 weeks of colonization, the animals were started on either a standard diet (STD) (Research Diets D12450B, the diet composed of 20%, 70%, and 10% calories from protein, carbohydrate, and fat respectively) or the HFD (Research Diet D12492, composed of 20%, 20%, and 60% calories from protein, carbohydrate, and fat, respectively) purchased from Research Diets Inc. (New Brunswick, NJ, USA). This experimental design resulted in four groups: the HL feces inoculated in B6 mice fed the standard diet (HL-STD), the HL feces inoculated into B6 mice fed the high-fat diet (HL-HFD), the NASH patients’ feces inoculated into B6 mice fed the standard diet (NASH-STD), and the NASH patients’ feces inoculated into B6 mice fed the high-fat diet (NASH-HFD). After switching to the assigned diet, the mice were weighed weekly for 16 weeks and then euthanized by asphyxiation with 95% CO_2_ without fasting. Livers were extracted and fixed in 10% neutral buffered formalin for 24 h and next subjected to histopathological analysis or immunohistochemical staining. For preparation of frozen sections, tissues were fixed with Tissue Embedding Medium Compound (Tissue-Tek O.C.T. Compound, Sakura Finetek, Torrance, CA, USA), and rapidly frozen over a dry ice and hexane slush (−73 °C) and stored at −80 °C until sectioning on a cryostat at −20 °C. For liver TG assay or gene expression analysis, tissues were stored in liquid nitrogen.

### 2.3. Clinical Chemistry

Whole blood was collected by cardiac puncture and centrifuged at 2600× *g* for 10 min at 4 °C. Serum was immediately stored at −80 °C until analysis of AST, ALT, GLU, TG, T-CHO, high density lipoprotein-cholesterol (HDL-C), and nonesterified fatty acids (NEFAs) on an automated analyzer (HITACHI 7080, Hitachi, Tokyo, Japan).

### 2.4. Histopathological Evaluation

Livers were fixed in 10% neutral buffered formalin for 1 day, dehydrated, embedded in paraffin, cut into 4-μm slices, and stained with hematoxylin and eosin (H&E) for histological examination.

### 2.5. Oil Red O Staining

Fresh liver tissue was embedded in Tissue-Tek 4583 OCT compound (Sakura Finetek, Torrance, CA, USA). Tissue was sectioned at 4 μm on a Universal Microtome Cryostat (Leica CM3050S, Leica Microsystems, Nussloch GmbH, Nussloch, Germany) and processed for examination of fat accumulation by Oil Red O staining. The liver tissues were fixed with 10% neutral buffered formalin for 10 min, stained with isopropanol for 5 min, then stained with fresh 60% Oil Red O working solution for 7 min, followed by staining with 85% isopropanol for 3 min and counterstaining with hematoxylin for 2 min.

### 2.6. Liver TGs

The liver samples weighed ~50 mg and were homogeneous. The liver TG concentration of each sample was determined by means of the Triglyceride Colorimetric Assay Kit (Cayman, Ann Arbor, MI, USA).

### 2.7. Limulus Amebocyte Lysate Test

To assay endotoxin levels, serum samples were collected aseptically, then diluted 1:5 with the Limulus Amebocyte Lysate (LAL) reagent solution in a nonpyrogenic tube by means of a commercially available Pyrochrome Limulus Amebocyte Lysate Kit (Associates of Cape Cod, Falmouth, MA, USA). *E. coli* O113:H10 served as the standard; a standard series ranging from 0.04 to 1.28 EU/mL was used to evaluate the concentration of endotoxin. Standards, samples, and a negative control were heated for 15 min at 75 °C, then added into wells of a 96-well plate with 50 μL of pyrochrome already present in each well, mixed for 30 s, and incubated at 37 °C for 25 min. To stop the reaction, 25 μL of 50% acetic acid was added. Optical density (OD) was determined at 405 nm on a Multiskan™ GO ELISA reader (Thermo Scientific, Waltham, MA, USA). Calculations were performed in endotoxin testing with concentrations expressed in EU/mL.

### 2.8. Serum Levels of Tnf-α, IL-6, Mcp1, and Insulin

Mouse Serum Adipokine LINCOplex Kit (Millipore, Billerica, MA, USA) was applied to quantify serum tumor necrosis factor alpha (TNF-α), interleukin 6 (IL-6), monocyte chemotactic protein 1 (Mcp1), and insulin. The assays were conducted using the Luminex technology in accordance with the manufacturer’s protocol.

### 2.9. Quantitative Real-Time Reverse-Transcription PCR (qRT-PCR)

RNA from the liver was isolated with the RNeasy Mini Kit (Qiagen, MA, USA). mRNA expression levels of *Tnf-*α, *Il6*, *Mcp1*, Toll-like receptor 2 (*Tlr2*), Toll-like receptor 4 (*Tlr4*), peroxisome proliferator-activated receptor gamma (*Ppar-γ*), and β-actin (*Actb*) were measured by qRT-PCR (for primer sequences used in the study see Table S2.) qRT-PCRs were carried out on LightCycler 1.5 (Roche, Mannheim, Germany) with the Light Cycler TaqMan Master Kit (Roche, Mannheim, Germany). Preincubation was performed at 94 °C for 10 min, followed by 40 cycles of denaturation at 94 °C for 10 s, then annealing at 60 °C for 30 s, and extension at 72 °C for 1 s. LightCycler^®^ Software 3.5 (Roche, Mannheim, Germany) was used to perform relative quantification, with β-actin as internal control. 

### 2.10. Gut Bacterial Composition

To investigate the composition of the gut microbiota of humanized gnotobiotic mice (groups HL-STD, HL-HFD, NASH-STD, and NASH-HFD), fresh fecal samples from three individuals were collected at 4 weeks after oral inoculation and 16 weeks on the designated diets (indicated as Week 0 and Week 16, respectively). Each fresh fecal sample was first normalized to 20 mg/mL under anaerobic conditions, 10-fold dilution was performed under anaerobic conditions, and 0.05-mL samples were inoculated onto each of 5 nonselective and 13 selective agar media. Isolated bacteria were identified by analyzing colony and cell morphology, aerobic growth, and spore formation as well as by Gram staining. The bacterial counts per sample for each bacterial group were calculated and expressed as a log_10_ (colony-forming units (CFU)/g) [16,17,18].

### 2.11. Statistical Analysis

All the data except the histopathologic score are presented as mean ± standard deviation (SD). SPSS ver. 18 (PASW Statistics for Windows, ver. 18.0, SPSS, Chicago, IL, USA) was used to analyze differences among groups. To compare values obtained from these groups, one-way analysis of variance was carried out, followed by Tukey’s post hoc test. Data with *p* < 0.05 were considered statistically significant. A two-way analysis of variance (ANOVA) was employed to compare multiple groups with a least significant difference (LSD) test. The significance of differences between histopathology scores was evaluated by the two-tailed Mann–Whitney *U* test. Data with *p* < 0.05 were regarded as statistically significant.

## 3. Results

### 3.1. Effects of the Gut Microbiota on Body, Liver, and Epididymal Fat Weight in Mice

In the HFD group, after the 16-week dietary intervention, the absolute epididymal fat weight and relative epididymal fat weight (percent of terminal body weight) were significantly higher in the NASH-HFD group than in the HL-HFD group. In the STD groups, the average of absolute epididymal fat weight and relative epididymal fat weight were higher in group NASH-STD than in the HL-STD group. Nonetheless, no significant differences in the terminal body weight, liver weight, and relative liver weight (percent of terminal body weight) were observed between groups NASH-HFD and HL-HFD (Table 1). 

### 3.2. Changes in Clinical Biochemistry Parameters as a Result of Colonization by Different Human Gut Microbiota

**Figure 1:**
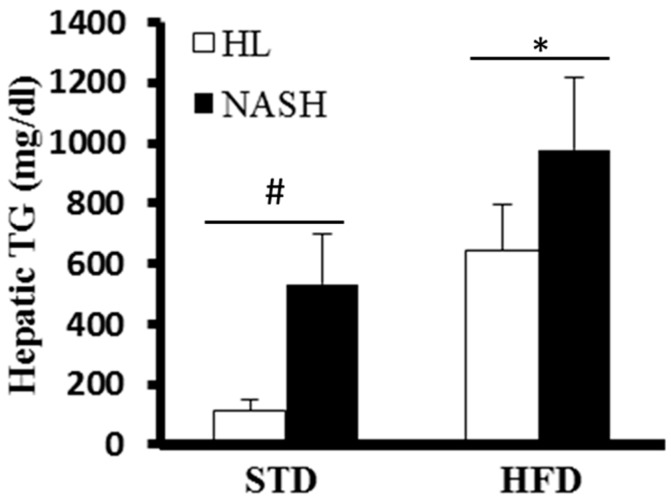
Concentration of hepatic triglycerides (TGs) in different groups. The hepatic TG level of the “inoculation with bacteria from nonalcoholic steatohepatitis patients and standard diet” (NASH-STD) group was 4.6-fold higher than that of the “inoculation with bacteria from healthy humans and standard diet” HL-STD group; hepatic TG levels in the NASH-high fat diet (HFD) group were 1.5-fold higher than those of the HL-HFD group. Data are presented as mean ± SD. HL: healthy humans; NASH: NASH patients; STD: standard diet; HFD: high-fat diet; TG: triglycerides; ^#^ *p* < 0.05 compared with STD-fed HL group; * *p* < 0.05 compared with HFD-fed HL group.

The clinical biochemistry profiles are presented in Table 2. Serum ALT and AST levels were significantly higher in the NASH-HFD group than in the HL-HFD group. Moreover, a similar trend was seen in the NASH-STD group compared with HL-STD group. Serum glucose was significantly higher in the NASH-HFD group compared with the other groups. The serum concentrations of T-CHO, TG, and HDL-C were higher in the HFD-fed mice than in STD-fed mice. The NASH-HFD group showed marked accumulation of hepatic TGs (1.5-fold, *p =* 0.0021) in the liver tissues, as compared with the HL-HFD group. Of note, the levels of liver TGs in the NASH-STD group were 4.6-fold higher than those in the HL-STD group (Figure 1).

### 3.3. Liver of the NASH-HFD Mice Showed a More Advanced Stage of Steatohepatitis

**Figure 2:**
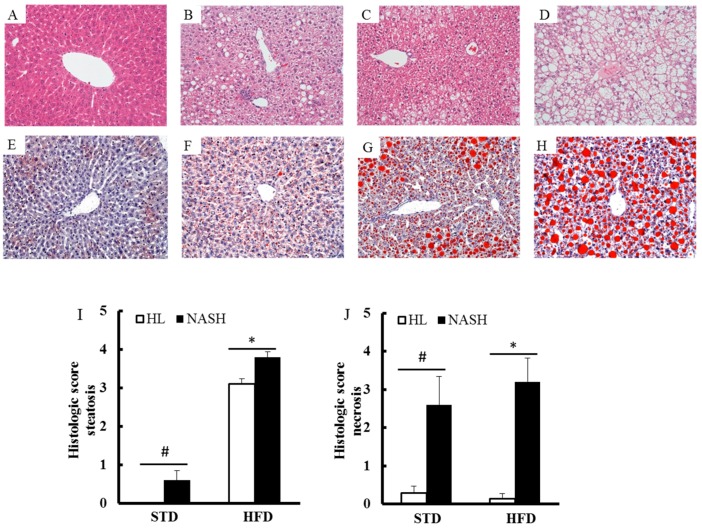
Histological evaluation of hepatic steatosis and necrosis and effects of the gut microbiota on hepatic lipid accumulation: (**A**) no significant alteration of the liver was seen in the HL-STD group; (**B**) only mild steatosis and moderate necrosis were observed in the NASH-STD group; (**C**) mild to moderate steatosis was seen in the HL-HFD group; (**D**) severe steatosis and multifocal necrosis were detected in the NASH-HFD group; (**E**) very few lipid droplets in the HL-STD group; (**F**) the modest amounts of lipid droplets in the NASH-STD group; (**G**) the mild to moderate amounts of lipid droplets in the HL-HFD group; (**H**) the abundant lipid droplets in the NASH-HFD group; (**I**) determination of histological steatosis scores; and (**J**) determination of histological necrosis scores. HL: healthy humans; NASH: NASH patients; STD: standard diet; HFD: high-fat diet. Hematoxylin and eosin (H&E) and Oil Red O staining (200×); ^#^ *p* < 0.05 compared with the STD-fed HL group; * *p* < 0.05 compared with the HFD-fed HL group.

Histological grading and staging system for liver steatosis and necrosis were based on the study by Brunt et al. [19]. The NASH-HFD group showed a more advanced stage of steatosis (macro- and micro-vesicular) and multifocal necrosis. Moreover, the mild to moderate steatosis but not necrosis was seen in the HL-HFD group as compared with in the NASH-HFD group. Only mild steatosis and moderate necrosis were observed in the NASH-STD group (Figure 2A–D). In contrast, there were no lipid droplets or multifocal necrosis in the HL-STD group (Figure 2E–H). The average scores for steatosis were 3.8 ± 0.1, 3.1 ± 0.1, 0.6 ± 0.3, and 0 ± 0 in the NASH-HFD, HL-HFD, NASH-STD, and HL-STD groups, respectively (Figure 2I). The mean necrosis score was 3.2 ± 0.6 for the NASH-HFD group, which was greater than the score of 0.1 ± 0.1 in the HL-HFD group (*p* < 0.01; Figure 2J). Consistent with the results on steatosis on H&E staining, the Oil Red O-stained sections revealed a higher degree of stained lipid droplets in the NASH-HFD mice than in the HL-HFD or NASH-STD mice. Furthermore, very few lipid droplets were observed in the HL-STD animals.

### 3.4. Serum Levels of Cytokines and Endotoxin Are Significantly Elevated in NASH-HFD Mice

**Figure 3:**
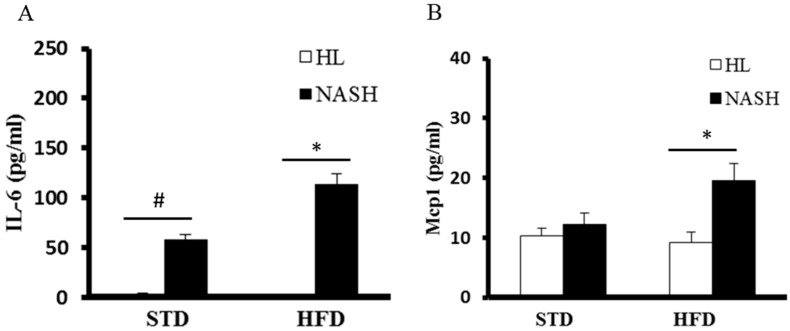
Serum concentrations of IL-6 and *Mcp1* in HL and NASH groups of mice fed STD or HFD for 16 weeks: (**A**) serum IL-6 level; and (**B**) serum Mcp1 level. Data are presented as mean ± standard deviation (SD). HL: healthy humans; NASH: NASH patients; STD: standard diet; HFD: high-fat diet; IL-6: interleukin-6; *Mcp1*: monocyte chemoattractant protein 1; ^#^ *p* < 0.05 compared with the STD-fed HL group; * *p* < 0.05 compared with the HFD-fed HL group.

**Figure 4:**
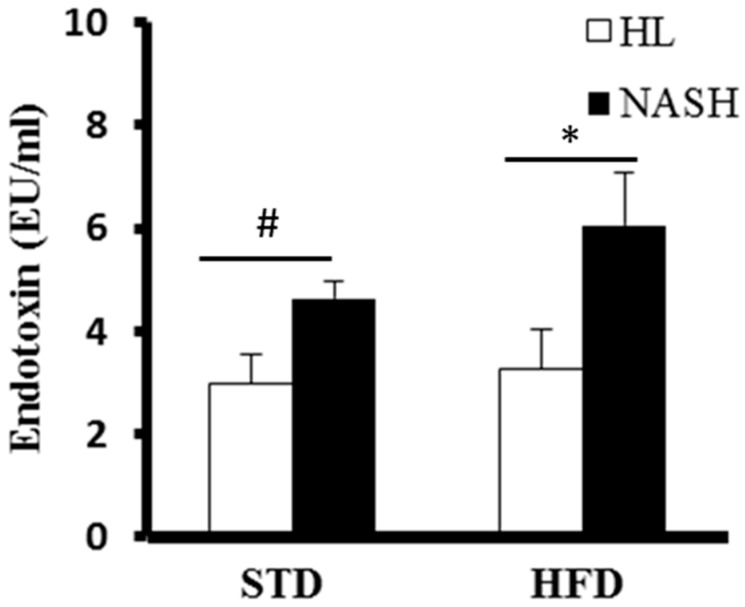
Serum concentrations of endotoxin in HL and NASH mice fed STD or HFD for 16 weeks. Data are presented as mean ± standard deviation (SD). HL: healthy humans; NASH: NASH patients; STD: standard diet; HFD: high-fat diet. ^#^ *p* < 0.05 compared with the STD-fed HL group; * *p* < 0.05 compared with the HFD-fed HL group.

The serum level of IL-6 was significantly higher in the NASH-HFD group (113.7 ± 10.8 pg/mL) compared to the NASH-STD group (58.4 ± 4.2 pg/mL); however, it was not detected in the serum of HL-mice, regardless of whether they were fed HFD or STD (Figure 3). The serum levels of *Mcp1* of the NASH-HFD group were 2.1-fold higher than those of the HL-HFD group; however, there were no statistically significant differences between groups HL-STD and NASH-STD. Serum *Tnf-α* was not detected in any group. The serum endotoxin levels were 2.96 ± 0.59, 4.64 ± 0.39, 3.27 ± 0.78, and 6.05 ± 1.04 EU/mL in groups HL-STD, NASH-STD, HL-HFD, and NASH-HFD, respectively. Our data indicated that the serum levels of endotoxin were significantly elevated in NASH-HFD mice (Figure 4).

### 3.5. Analysis of Gene Expression by Real-Time PCR

**Figure 5:**
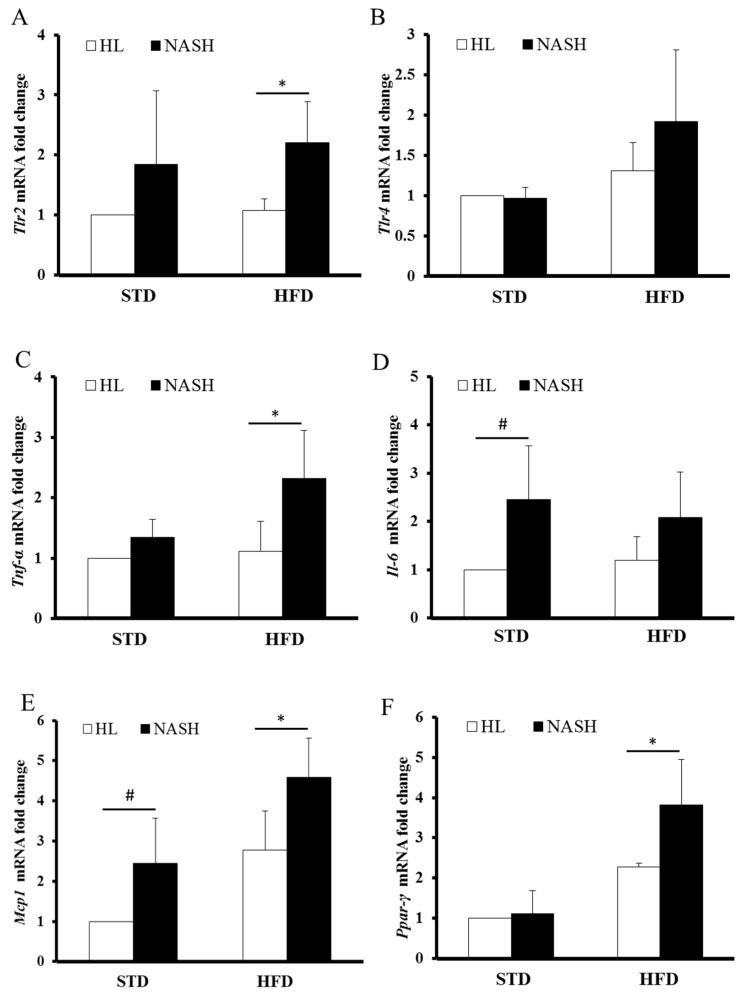
The gene expression levels of liver tissue in the HL and NASH mice fed STD or HFD; (**A**) *Tlr2* gene expression; (**B**) *Tlr4* gene expression; (**C**) *Tnf-α* gene expression; (**D**) *IL-6* gene expression; (**E**) *Mcp1* gene expression; and (**F**) *Ppar-γ* gene expression. Data are presented as mean ± standard deviation (SD). HL: healthy humans; NASH: NASH patients; STD: standard diet; HFD: high-fat diet; *Tlr2*: Toll-like receptor 2; *Tlr4*: Toll-like receptor 4; *Tnf-α:* tumor necrosis factor *α*; *Mcp1*: monocyte chemoattractant protein 1; *Ppar-γ*: peroxisome proliferator-activated receptor-γ. ^#^ *p* < 0.05 compared with the STD-fed HL group; * *p* < 0.05 compared with the HFD-fed HL group.

*Tlr2* expression levels were elevated in the NASH group compared to the HL- group regardless of the diet (Figure 5A). The expression level of *Tlr4* was the highest in the NASH-HFD group (Figure 5B). The expression of *Tnf-α* was significantly higher in the NASH-HFD mice and slightly increased in the NASH-STD group, and showed no differences between the HL-STD and the HL-HFD groups (Figure 5C). The mRNA expression of *Il6* was higher in NASH mice than in HL mice, for both STD- and HFD-fed groups (Figure 5D). As for the *Mcp1* expression, the HL-STD group had significantly lower levels than did the other groups, with group NASH-HFD showing the highest levels among them (Figure 5E). *Ppar-γ* mRNA expression was higher in the NASH group, with the NASH-HFD group showing the highest expression (Figure 5F).

### 3.6. Analysis of Gut Microbiota Composition

**Figure 6:**
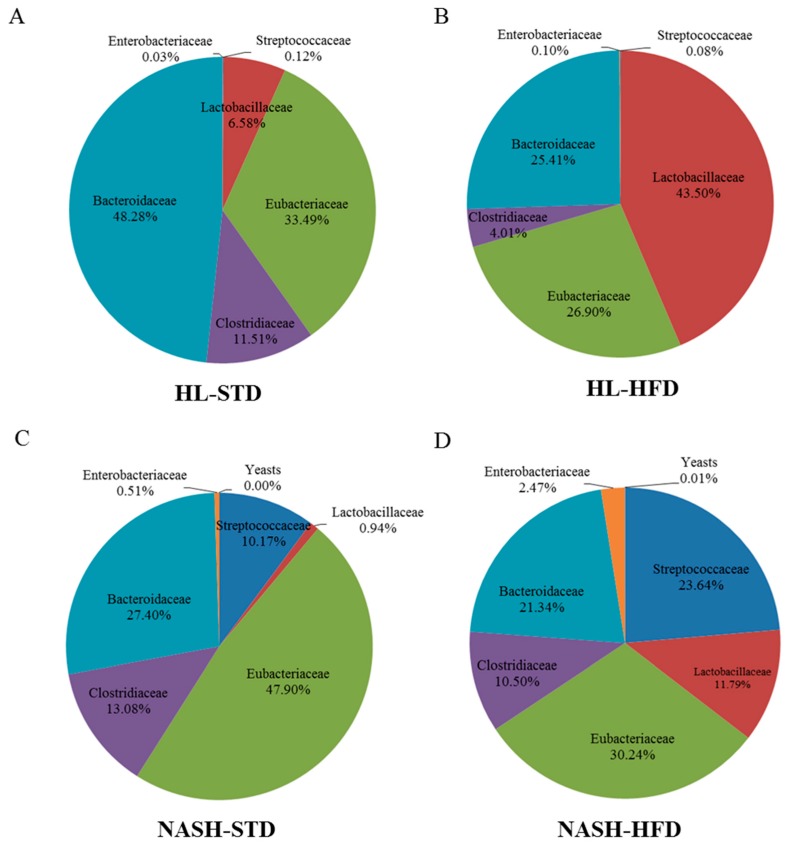
Family distribution of the gut microbiota of HL and NASH mice fed STD or HFD: (**A**) HL-STD, mice fed STD for 16 weeks; (**B**) HL-HFD, mice fed HFD for 16 weeks (**C**) NASH-STD, mice fed STD for 16 weeks; and (**D**) NASH-HFD, mice fed HFD for 16 weeks, (*n* = 3, respectively). HL: healthy humans; NASH: NASH patients; STD: standard diet; HFD: high-fat diet.

The *Firmicutes* and *Bacteroidetes* were the two most abundant phyla in the gut microbiota. The relative abundance of *Firmicutes* (including *Lactobacillales*, *Streptococcaceae*, *Eubacteriaceae*, and *Clostridiaceae*) was significantly higher in groups NASH-HFD, HL-HFD, and NASH-STD than in the HL-STD group; however, the relative abundance of *Bacteroidetes* was the highest in the HL-STD group (Table 3, Figure 6). The relative abundance of the phylum *Proteobacteria* (*Enterobacteriaceae*) was significantly higher in the NASH-HFD group than in the HL-HFD group. When we analyzed gut microbiota composition at the family level, the relative abundance of *Streptococcaceae* was higher in the NASH-HFD than NASH-STD group with no significant changes observed in groups HL-STD and the HL-HFD. A significant increase in the number of *Lactobacillaceae* cells was found in HL-HFD mice compared with the other groups. No significant difference in *Clostridiaceae* was observed among groups HL-STD, NASH-STD, and NASH-HFD. Of note, *Clostridiaceae* were less abundant only in the HL-HFD group. Moreover, *Enterobacteriaceae* were upregulated only in the NASH-HFD group. Taken together, our data suggested that the pathological progression of NAFLD may be associated with the relative abundance of these intestinal microbes.

## 4. Discussion

Some studies indicate that the gut microbiota plays an important role in the regulation of host metabolism homeostasis, innate immunity, development of insulin resistance, and progression of obesity [20,21,22]. Several studies have used GF rodents to elucidate the intricate and complex interactions among host metabolism, intestinal flora, and progression of obesity, diabetes, and cardiovascular diseases [22,23,24]. In addition, the gut microbiota was recently shown as an environmental factor that may promote metabolic diseases such as NAFLD and NASH [20,23]. In the present study, we employed a novel humanized gnotobiotic mouse model and demonstrated that colonization with fecal bacteria from NASH patients along with HFD feeding can aggravate disease progression of NAFLD. These phenotypic variations may correlate with an increase in the levels of inflammatory cytokines (IL-6, *Mcp1*, and *Tnf-α*), TLR expression enrichment (*Tlr2* and *Tlr4*), insulin resistance (insulin and *Ppar-γ*), and an alteration of gut microbiota composition (a decrease in the relative abundance of *Bacteroidaceae* and an increase in the relative abundance of *Streptococcaceae*). In addition, gnotobiotic mice colonized with fecal bacteria from healthy humans and fed HFD showed only simple steatosis without necrosis in the liver. The hepatic morphological and functional changes may correlate with a decreased proportion (abundance) of *Clostridiaceae* and increased proportion of *Lactobacillaceae.*

Our results show that there were no differences in the body weight and liver weight between groups HL-STD and NASH-STD or between groups HL-HFD and NASH-HFD. Of note, the weight of epididymal fat in mice inoculated with the NASH human fecal mixture, regardless of whether the mice were fed STD or HFD, was significantly higher than that of mice inoculated with the HL fecal mixture. In one study, Backhed and coworkers reported that the colonization of GF mice with microbiota from conventionally raised animals produces an increase in body fat [25]. Turnbaugh and colleagues reported that the obesity-associated microbiota has an increased ability to harvest energy from the diet [22]. Hence, colonization of GF mice with the “obesity-related microbiota” resulted in a significant increase in total body fat relative to GF mice colonized with a “thinness-related microbiota” [22]. Our results confirmed that inoculation of the gut microbiota from NASH patients in conjunction with high dietary fat content can indeed increase the accumulation of body and epididymal fat and aggravate the severity of NAFLD in the humanized gnotobiotic mice. These data suggest that the microbiota by itself may directly or indirectly cause an increase in weight and fat gain. Furthermore, in our study, regardless of whether STD or HFD was administered, the gut microbiota of mice inoculated with HL feces consisted of a greater proportion of *Bacteroidetes* than that of mice inoculated with the feces of NASH patients. The opposite effect was observed for *Firmicutes*. Research has shown that the relative abundance of *Bacteroidetes* is lower and that of *Firmicutes* is higher in obese mice compared to their leaner counterparts [26]. The level of *Bacteroidetes* is significantly lower in obese subjects as compared with normal-weight subjects [13]. These studies suggest that interventions targeting the metabolism-associated activity of gut microbiota might be effective in treating obesity and the associated metabolic disorders such as NAFLD and NASH.

Recent evidence from humans and from animal models has also linked gut microbiota to the pathological progression of NAFLD/NASH via the gut–liver axis [27,28]. In the present study, ALT activity was significantly higher in mice inoculated with NASH feces than in those inoculated with HL feces. These results are not consistent with the cirrhosis patient severity, and might not correlate with the ALT levels according to recent reports [29].

Blood endotoxin concentration and hepatic TG showed similar results. Hepatic steatosis, inflammation, and fibrosis are key factors in the pathogenesis of NASH [5,30]. In this study, the results revealed that severity of micro- and macrosteatosis in the NASH-HFD group is higher as compared with the HL-HFD group. Moreover, mild to moderate necrosis was observed only in the NASH-HFD group. Histological characteristics of present mouse model, such as (macro- or microvesicular) steatosis and multifocal necrosis were similar to those of human NAFLD. However, other histological features were absent (e.g., pigmented macrophages, megamitochondria, and Mallory–Denk bodies). These findings are consistent with a previous report that indicated the above lesions would not appear in mice [4]. In contrast, in the histological analyses, no liver fibrosis was observed in any of the animals. Recent studies indicate that the inflammasome may perform an important function in NAFLD and NASH progression [31] and pointed out that the intestinal microflora can regulate activities of the inflammasome [2,32,33]. Therefore, we suggest that inoculated NASH gut microbiota causing severe NAFLD might be related to inflammasome activation, and further experiments are needed for verification. 

Several reports point to strong correlations between the gut microbiota and inflammatory cytokines and the contribution to the progression of NAFLD [23,27]. The TLR family of pattern recognition receptors is critical for host defense against invading pathogens [32]. In the present work, the serum level of inflammatory cytokine IL-6 was higher in both the human NAFLD patients and the NAFLD feces-inoculated gnotobiotic animals regardless of dietary fat content [34,35]. Recently, Mas and coworkers found that the severity of diet-induced NASH is lower in *Il6*-deficient mutant mice compared to controls [36]. Our results are consistent with those in other reports, and the serum level of IL-6 may be associated with colonization by human NASH patients’ fecal bacteria. The serum *Mcp1* protein levels and mRNA levels of *Mcp1* were higher in the NASH-HFD group than in others. The *Mcp1*-deficient mice fed an HFD show reduced insulin resistance and hepatic steatosis [37]. On the other hand, mice overexpressing *Mcp1* in adipose tissues show increased insulin resistance and hepatic TG levels [38]. Our findings are in agreement with other reports that *Mcp1* may promote liver fat accumulation and the associated inflammation.

Our data suggest that the mRNA expression levels of *Tlr2* and *Tlr4* are higher in the NASH-HFD group than in the other groups. Several animal models have revealed that TLR deficiency-associated changes in the gut microbiota composition are related to exacerbated hepatic steatosis and inflammation [2]. Another study indicates that gut microbiota dysbiosis can cause an influx of *Tlr4* and *Tlr9* agonists into the portal circulation, leading to enhanced hepatic *Tnf-α* expression that drives NASH progression [2,37]. *Tlr4* is the receptor for endotoxin, which is an important mediator of liver inflammation associated with NAFLD and hepatocellular carcinoma [39,40]. A close association between NAFLD and increased levels of circulating endotoxin has been observed in obese mice and humans [41]. Furthermore, Ye and colleagues demonstrated that *Tlr4* is essential for hepatic fat deposition and NASH development [42]. Hence, gut microbiota colonization from NASH feces in conjunction with the HFD might drive NAFLD progression. Moreover, the inoculation with intestinal flora from healthy people and feeding the HFD did not induce an inflammatory reaction but steatosis could still be seen. Research suggests that immune modulation of the gut microbiota induces symptoms of metabolic syndrome in *Tlr2* knockout mice [43]. Conversely, we also found that expression of the *Tlr2* gene was upregulated in the in the NASH-HFD group. We suspected that these alternations might due to changes of intestinal microbiota composition. These data indicate that inflammatory cytokines, *Tlr2*- and *Tlr4*-mediated pathways crucially contribute to the progression of NAFLD/NASH.

Some literature data indicate that, in rats fed a fructose-rich diet, the development of metabolic syndrome directly correlates with variations of the gut concentration of specific bacterial taxa [44]. In our study, the blood glucose level in the NASH-HFD group was significantly higher than that of the HL-HFD group; however, the opposite effect was observed for insulin. Several research groups demonstrated a correlation between type 2 diabetes and obesity [20,45]. Membrez and coworkers used ob/ob mice to show that modulation of the gut microbiota via antibiotics reduces hepatic steatosis and improves glucose sensitivity [46]. Furthermore, we observed that the insulin levels were lower in the NASH-HFD group than in the HL-HFD group. A similar result was obtained for the STD-fed group. Our findings indicate that the colonization with fecal bacteria from HL should have a greater relation with obesity or NAFLD (simple steatosis type) induction. In contrast, the feces from NASH may correlate with NAFLD (steatohepatitis type). Cryer reported that an increase in serum glucose concentration induces an increase in insulin secretion by β-cells of the pancreas; however, β-cell failure did not result in a decrease in β-cell insulin secretion [47]. Furthermore, Satoch and coworkers demonstrated that hepatic steatosis is induced by an increase in incorporation of fatty acids into the liver via increased *Ppar-γ* expression in the liver of insulin-resistant mice [48]. The results of the present study are consistent with these findings. In contrast, mice inoculated with fresh feces from a healthy person showed only glucose intolerance. Therefore, the imbalance of gut microbiota might correlate with insulin resistance and contributed to NAFLD severity.

In recent years, experimental and clinical studies have shown a pathogenic association between the gut microbiota and NAFLD. Le Roy and coworkers demonstrated that gut microbiota contributes to the development of NAFLD independently of obesity [27]. The different bacterial groups that we investigated in this study are affiliated with three phyla: *Bacteroidetes*, *Firmicutes*, and *Proteobacteria*, which are crucial for gut homeostasis and are believed to be involved in NAFLD. Our results indicate a higher abundance of the phylum *Firmicutes* and a lower abundance of the phylum *Bacteroidetes*, as well as *Proteobacteria* in the NASH-HFD group when compared with group HL-STD. These results are in agreement with those of Sefcikova and other researchers [48,49,50,51]. Additionally, *Firmicutes*, particularly *Streptococcaceae*, have been noticed in the NASH-HFD group but not in the NASH-STD group. In contrast, they were not observed in HL groups. As compared to healthy subjects, patients with NAFLD also had an increased percentage of bacteria from the families *Streptococcaceae* and *Enterobacteriaceae*, both known to induce persistent inflammation in the intestinal mucosa and to be associated with IBD [52,53]. A significant increase in the number of *Lactobacillaceae* was found in group HL-HFD. Other studies that compared obese patients with healthy controls uncovered an increase in cell counts of genus *Lactobacillus* and a decrease in the family *Ruminococcaceae* in obese patients [54,55]. Regarding the association with *Lactobacillus*, it is surprising, because several species from this genus are frequently used as probiotics. *Lactobacillus* is lactic acid bacteria that can suppress pathogens, enhance epithelial barrier function, and modulate immune responses [56]: actions that seem to protect from the pathogenesis of NAFLD and/or NASH. In contrast, *Lactobacillus* may be associated with the production of volatile organic compounds such as acetate and ethanol [57], which may be important for the pathogenesis of obesity and NAFLD [53]. Patients with NASH were recently shown to have lower abundance of bacteria belonging to the phylum *Bacteroidetes* (family: *Bacteroidaceae*) compared to subjects with simple steatosis and healthy individuals as shown by qPCR [57]. In addition, there are gender differences in the results on Western-diet-induced steatosis severity [58]. Additionally, Jena and coworkers reported that the Western-diet-fed male Farnesoid X Receptor (FXR) knockout mice have severer steatohepatitis as compared to male wild-type mice. The antibiotic treatment might reduce hepatic inflammation in each mouse [58,59]. Especially, *Lactococcus*, *Lactobacillus*, and *Coprococcus* decrease the liver inflammation [58]. These results revealed the relationship between Western diet and gut microbiota might play important roles in NAFLD and NASH.

## 5. Conclusions

The results presented here provided evidence that composition of the intestinal microbiota may interfere with NAFLD and/or NASH progression through multiple inflammation reactions. As far as we know, this is the first report of exacerbation of NAFLD in the HFD-fed humanized gnotobiotic mice after direct colonization with fecal bacteria from NASH patients, but not healthy people. In addition, we found complex and cooperative effects of Tls, inflammatory cytokines, and host gut microbiota on NAFLD parameters. Taken together, our findings may lead to novel strategies (such as colonization with fecal bacteria from NASH patients) to investigate NAFLD pathogenesis.

## Figures and Tables

**Table 1:** Terminal body weight and liver and fat pad weights of HL and NASH mice fed STD or HFD.

Characteristic					*p* Values for Two-Way ANOVA		
	STD		HFD		Main Effect of Microbiota	Main Effect of Diet	Interaction (M × D)
	HL	NASH	HL	NASH			
Terminal body weight (g)	30.7 ± 2.8	31.6 ± 2.0	48.1 ± 3.0	48.6 ± 3.0	0.532	<0.0001	0.852
Absolute liver weight (g)	1.4 ± 0.2	1.4 ± 0.1	2.6 ± 0.3	2.4 ± 0.8	0.621	<0.0001	0.674
Relative liver weight (percent of terminal body weight)	4.5 ± 0.3	4.3 ± 0.2	5.2 ± 0.3	4.9 ± 1.5	0.394	0.049	0.747
Absolute epididymal fat weight (g)	0.9 ± 0.2	1.3 ± 0.2 ^#^	1.9 ± 0.2	2.7 ± 0.5 *	<0.0001	<0.0001	0.077
Relative epididymal fat weight (percent of terminal body weight)	2.8 ± 0.4	4.1 ± 0.5 ^#^	3.9 ± 0.3	5.7 ± 1.4 *	<0.0001	0.0002	0.326

HL: healthy humans; NASH: NASH patients; STD: standard diet; HFD: high-fat diet; ANOVA: a two-way analysis of variance; M: microbiota; D: diet. Values are mean ± standard deviation (SD) for *n* = 10 mice in each group; ^#^ *p* < 0.05 compared with STD-fed HL group; * *p* < 0.05 compared with the HFD-fed HL group.

**Table 2:** Serum chemistry and insulin levels in the HL and NASH groups fed STD or HFD.

					*P* Values for Two-Way ANOVA		
Characteristic	STD		HFD		Main Effect of Microbiota	Main Effect of Diet	Interaction (M × D)
	HL	NASH	HL	NASH			
ALT (U/L)	14.7 ± 2.8	53.1 ± 25.5	138.3 ± 58.7	230.7 ± 83.1 *	0.009	<0.0001	0.248
AST (U/L)	78.2 ± 14.5	161.0 ± 23.9 ^#^	127.8 ± 30.2	218.2 ± 67.0 *	0.0001	0.007	0.832
Glucose (mmol/L)	15.6 ± 0.80	16.5 ± 0.87	15.7 ± 2.46	20.7 ± 0.89 *	0.017	0.078	0.085
T-CHO (mmol/L)	3.67 ± 0.78	3.01 ± 0.8	6.22 ± 0.32	6.48 ± 0.91	0.531	<0.0001	0.146
TGs (mmol/L)	0.63 ± 0.26	0.62 ± 0.12	0.81 ± 0.21	0.98 ± 0.24	0.413	0.006	0.330
HDL-C (mmol/L)	2.96 ± 0.26	2.47 ± 0.62 ^#^	4.05 ± 0.19	3.97 ± 0.37	0.087	<0.0001	0.218
NEFAs (mmol/L)	0.7 ± 0.2	0.7 ± 0.2	1.4 ± 0.1	1.2 ± 0.2	0.373	<0.0001	0.135
Insulin (μU/mL)	74.9 ± 24.1	25.4 ± 3.7 ^#^	171.3 ± 36.1	99.9 ± 19.9 *	0.0001	<0.0001	0.368
HOMA-IR	52.8 ± 9.3	18.4 ± 2.7 ^#^	119.4 ± 28.1	88.9 ± 20.6	0.0058	<0.0001	0.8473

HL: healthy humans; NASH: NASH patients; STD: standard diet; HFD: high fat diet; ALT: alanine aminotransferase; AST: aspartate aminotransferase; GLU: glucose; TGs: triglycerides; T-CHO: total cholesterol; HDL-C: high density lipoprotein-cholesterol; NEFAs: nonesterified fatty acids; HOMA-IR: homeostasis model assessment for insulin resistance; ANOVA: a two-way analysis of variance; M: microbiota; D: diet. Values are mean ± standard deviation (SD) for *n* = 10 mice in each group; ^#^ *p*< 0.05 compared with STD-fed HL group; * *p* < 0.05 compared with the HFD-fed HL group.

**Table 3:** Changes in the gut microbiota of HL and NASH mice fed STD or HFD.

Characteristic	STD		HFD	
	HL	NASH	HL	NASH
*Streptococcaceae*	7.3 ± 0.3	9.1 ± 0.4	7.1 ± 0.7	9.8 ± 0.1 *
*Lactobacillaceae*	9.1 ± 0.8	8.0 ± 0.3	9.8 ± 0.2	9.5 ± 0.6
*Eubacteriaceae*	9.8 ± 0.2	9.7 ± 0.4	9.6 ± 0.3	9.9 ± 0.1
*Clostridiaceae*	9.3 ± 0.1	9.2 ± 0.6	8.8 ± 0.4	9.5 ± 0.2 *
*Bacteroidaceae*	9.6 ± 0.5	9.5 ± 0.2	9.6 ± 0.6	9.8 ± 0.4
*Enterobacteriaceae*	6.7 ± 0.4	7.8 ± 0.8	7.2 ± 0.6	8.9 ± 0.9 *
*Yeasts*		5.4 ± 0.1 ^#^		6.6 ± 0.1 *
Total counts	10.2 ± 0.2	10.1 ± 0.2	10.2 ± 0.2	10.4 ± 0.1

Mean ± standard deviation (SD) of log values for *n* = 3 in each group. Counts of bacteria per gram of feces; ^#^ *p* < 0.05 compared with the STD-fed HL group; * *p* < 0.05 compared with the HFD-fed HL group. HL: healthy humans; NASH: NASH patients; STD: standard diet; HFD: high-fat diet.

## Supplementary Materials

The following are available online at www.mdpi.com/2072-6643/9/11/1220/s1, Table S1: Anthropometric and metabolic variables of donors, Table S2: Real-time PCR primers used in this study.
