# Supplementary material for: Nonalcoholic Fatty Liver Disease Is Exacerbated in High-Fat Diet-Fed Gnotobiotic Mice by Colonization with the Gut Microbiota from Patients with Nonalcoholic Steatohepatitis
Source: Nutrients. 2017 Nov 6;9(11):1220. doi: 10.3390/nu9111220 (PMC5707692; doi:10.3390/nu9111220)
Supplement: Supplementary file 1 [file nutrients-09-01220-s001.pdf]

Nonalcoholic Fatty Liver Disease Is Exacerbated in High-Fat Diet-Fed Gnotobiotic Mice by Colonization with the Gut Microbiota from Patients with Nonalcoholic Steatohepatitis

Table S1. Anthropometric and metabolic variables of donors.

Variables	Healthy donor (n = 10)	NASH donor (n = 10)
Weight (kg)	62.3±9.3	66.8±10.7
Height (cm)	165.0±7.7	161.1±8.9
BMI (kg/m^2)	22.8±1.5	25.6±2.7
ALT (U/L)	28.6±14.1	76.4±63.0
AST (U/L)	26.2±5.9	63.6±41.8
TG (mg/dl)	108.9±20.0	144.4±44.1
Chol (mg/dl)	189.2±18.2	192.3±18.4
Glu (mg/dl)	90.2±4.8	102.8±18.0

BMI: body mass index; TG: triglycerides; Chol: cholesterol; Glu: fasting glucose.

Table S2. Real-time PCR primers used in this study.

Gene (NCBI ID)	Orientation	Sequence (5′-3′)	UPL
TNF-α (NM_013693.2)	Forward	TGCCTATGTCTCAGCCTCTTC	49
	Reverse	GAGGCCATTTGGGAACTTCT	
IL-6 (NM_031168.1)	Forward	GCTACCAAACTGGATATAATCAGGA	6
	Reverse	CCAGGTAGCTATGGTACTCCAGAA	
MCP-1 (NM_011333.3)	Forward	CATCCACGTGTTGGCTCA	62
	Reverse	GATCATCTTGCTGGTGAATGAGT	
PPAR-γ (NM_011146.3)	Forward	TGCTGTTATGGGTGAAACTCTG	2
	Reverse	CTGTGTCAACCATGGTAATTTCTT	
TLR 2 (NM_011905.3)	Forward	GGGGCTTCACTTCTCTGCTT	50
	Reverse	AGCATCCTCTGAGATTTGACG	
TLR 4 (NM_021297.2)	Forward	GGACTCTGATCATGGCACTG	2
	Reverse	CTGATCCATGCATTGGTAGGT	
β-actin (NM_007393.3)	Forward	CTAAGGCCAACCGTGAAAAG	64
	Reverse	ACCAGAGGCATACAGGGACA	
